# Molecular Simulation of the Water Diffusion Behavior and Electronic Properties of Boron-Nitride-Composited Mineral Oil

**DOI:** 10.3390/molecules29184500

**Published:** 2024-09-22

**Authors:** Yang Wang, Wenchao Yan, Kunqi Cui, Chuanhui Cheng, Yuanyang Ren, Kai Wu

**Affiliations:** 1 School of Electronics and Information, Xi’an Polytechnic University, Xi’an 710048, China; ywchao@stu.xpu.edu.cn (W.Y.); cuikunqi_xpu@163.com (K.C.); 2 Xi’an Key Laboratory of Interconnected Sensing and Intelligent Diagnosis for Electrical Equipment, Xi’an Polytechnic University, Xi’an 710048, China; 3 Electric Power Research Institute, China Southern Power Grid, Guangzhou 510663, China; 4 State Key Laboratory of Electrical Insulation and Power Equipment, Xi’an Jiaotong University, Xi’an 710049, China; yyrenxjtu@126.com (Y.R.); wukai@xjtu.edu.cn (K.W.)

**Keywords:** BN nanoparticles, mineral oil, diffusion of water molecules, interfacial potential barriers, molecular simulation

## Abstract

Despite the fact that doping nanoparticles into insulating transformer oil has proven to be an effective method of enhancing its dielectric and electrical properties, it remains unclear how different types and surface conditions of nanoparticles may affect their dielectric and electrical properties. Therefore, the effect of doping various types of BN nanoparticles (nanosphere, nanotube, and nanosheet) in insulating mineral oil (MO) on the diffusion properties of water molecules and electrical properties across the BN/MO interface was investigated using molecular dynamics (MD) and Density Functional Theory (DFT) simulations. Our results show that different surface morphology and grafted functional groups in different types of BN nanoparticles have a significant impact both on the water diffusion behavior and the interfacial potential barrier across the interface between BN and MO. In the MO system directly doped by BN nanospheres, water diffusion behavior is not significantly restricted. However, grafting -NH_2_ polar groups onto the BN nanoparticle surface may significantly limit the diffusion behavior of water due to the strong attraction between the -NH_2_ polar groups and water molecules; the most significant effect is with nanospheres, followed by nanotubes and nanosheets. In terms of electrical properties across the interface between BN and MO, the h-BN surface (derived from BN nanosheets and nanotubes) acts as a trap for electrons in MO (−0.59 eV), while the c-BN surface (derived from BN nanospheres) acts as a potential barrier for electrons in MO (1.45 eV), and it is noteworthy that the presence of water molecules near the interface between BN and MO has little impact on the potential barriers. Advancing a fundamental understanding of the electrical and water diffusion properties of MO in correlation with the surface morphology of different types of nanoparticles is key to improving the insulation properties of oil-impregnated power transformers.

## 1. Introduction

Power transformers are essential components of power supply systems, and their insulation performance directly affects their safe and stable operation [1,2]. Insulation failure within power transformers is the main cause of transformer-related safety accidents [3,4]. During operation, oil–paper insulation in oil-impregnated power transformers is normally exposed to high temperatures and electric fields for extended periods, resulting in gradual insulation degradation. The oil–paper insulation deteriorates over time and generates impurities such as short-chain alkanes, gases, and water molecules. The combined effect of complicated operating environments (high voltage and temperature) and impurities within the insulation oil, such as water molecules, accelerates the cracking and deterioration of the oil–paper insulation system, which may eventually lead to insulation failure [5]. Additionally, water molecules, owing to their strongly polar nature, can distort the internal electric field and form “discharge bridges”, severely affecting the electrical performance and significantly reducing the safe service life of the transformer [6,7]. Therefore, maintaining good dielectric and insulating properties of insulating oil is fundamental to ensure the long-term reliable operation of power transformers.

Miniaturization of electrical equipment and advancements in ultra-high-voltage transmission technologies have imposed higher insulation requirements on insulating oils. Currently, the insulating properties of transformer oils can be improved by adding antioxidants, blending different types of insulating oils, and doping nanoparticles (NPs) [8,9,10]. However, Hao et al. [11,12] found that the amount of antioxidants in the oil must be strictly controlled, requiring many instruments and reagents for regular testing. Wang’s team [13] investigated the effect of various blending ratios on insulating oil performance; they found that the increase in vegetable oil percentage negatively affected the insulating properties of the blended oil. Since different insulating oils have different physicochemical properties, their mixing ratios directly affect their performance, which may lead to instability, compatibility issues, and increased challenges during monitoring and diagnosis.

With the development of nanotechnology, doping nanoparticles has become one of the most commonly used strategies to improve the insulation performance of transformer oil [14]. Researchers used Al_2_O_3_ nanoparticles to modify transformer insulating oils, and their results showed that the doped Al_2_O_3_ nanoparticles introduced a large number of electron traps into the oil, leading to a reduction in dielectric loss [15,16]. Various metal oxide nanoparticles were used to modify transformer oils in order to improve their insulating properties, including CuO, Fe_3_O_4_, and ZnO nanoparticles [17,18,19]. As one of the most commonly used nonmetallic oxide NPs, SiO_2_ nanoparticles were also used for improving the dielectric and insulation properties of insulating oil [20,21]. Boron nitride (BN) nanoparticles, with a low dielectric constant, small dielectric loss, and high thermal conductivity, are one of the promising nanoparticles used for improving the electrical and thermal properties of insulating polymers [22,23,24,25,26]. Hexagonal boron nitride (h-BN) and cubic boron nitride (c-BN) are thermodynamically stable structures that can be synthesized in various types, such as nanospheres, nanotubes, and nanosheets. Similar to other nanoparticles, the practical applications of BN nanoparticles are considerably limited by their high surface energy and strong tendency to agglomerate. Various surface modification methods have been developed to enhance their dispersion properties and the interfacial properties between BN nanoparticles and the matrix [27]. Many scholars have performed surface functionalization on the edges of BN nanoparticles and then doped them into the matrix material to improve their electrical and dielectric performances [22,23,28]. Jamirad’s study demonstrated that hydroxylated BN could enhance the interaction between nanoparticle–matrix interfaces and reduce the vibrational coupling effect [29]. With Lei’s team reporting the successful production of aminated BN by urea-assisted ball milling, aminated BN has become a reality, further accelerating its wide application in various composite materials [30]. It has been demonstrated by Bai et al. that aminated BN provides better thermal conductivity to polyimide/BN composite at higher filling mass fractions than hydroxylated BN [31]. Therefore, aminated BN nanoparticles could be considered as one of the promising candidates for improving the overall performance of insulating materials. Wu et al. [32] studied the interaction energy between h-BN and water by a theoretical electronic structure method, but did not apply it to study the influence of different types of BN and surface treatment, nor did they study the electrical properties.

In spite of the fact that BN nanoparticles have been proven to improve the electrical and dielectric properties of insulating polymers, it remains unclear at the molecular level how the different types and surface conditions of BN particles may affect the diffusion behavior of water molecules and the electrical properties across the interface. Therefore, in this work, the doping of various types of BN nanoparticles (nanospheres, nanotubes, and nanosheets) in insulating mineral oil (MO) with or without surface treatment was selected as the research object to evaluate the diffusion properties of water molecules and electrical properties across the BN/MO interface using molecular dynamics (MD) and Density Functional Theory (DFT) simulations. The study focuses on the significant differences between amination-modified and unmodified BN surfaces, particularly regarding the diffusion behavior of water molecules in the model and the interfacial potential barriers across the BN/MO interfaces.

## 2. Results and Discussion

### 2.1. Diffusion Behavior of Water Molecules in MO/BN Composite Models

#### 2.1.1. Diffusion Coefficients in Different BN-Doped MO Models

The diffusion coefficient (*D*) is a crucial physical parameter to quantify the extent of diffusion in a system, indicating the ability of a species to diffuse within its medium. *D* can be determined using the mean square displacement (*MSD*) curve, which describes the average distance that a particle travels from its initial position over time [33]. The corresponding expression is shown in Equation (1), which helps to determine whether diffusion, transport, or jumping occurs by analyzing the trajectory of the particle:

(1) $$MSD=\langle {\left|\vec{r_{i}}\left(t\right)-\vec{r_{i}}\left(0\right)\right|}^{2}\rangle$$

**Figure 1:**
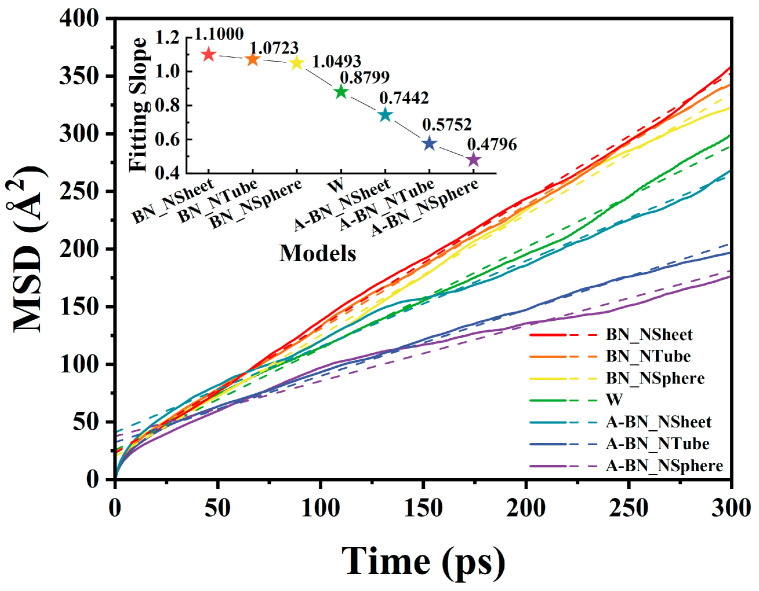
*MSD* curves of water molecules in MO doped with BN materials of different types.

Taking water molecules as an example, $\vec{r_{i}}\left(t\right)$ and $\vec{r_{i}}\left(0\right)$ represent the coordinate vectors of the molecule at time *t* and the initial moment, respectively. The “⟨ ⟩” notation indicates an average over all water-molecule-related values. Figure 1 shows the *MSD* curves for the MO/BN composite models along with their linear fits.

The *D* value of water molecules in oil can be calculated using the following equation [34]:

(2) $$D=\frac{1}{6N}\underset{t\to \infty }{\lim}\frac{d}{dt}\sum_{i=1}^{n}{\left|\vec{r_{i}}\left(t\right)-\vec{r_{i}}\left(0\right)\right|}^{2}=\frac{a}{6}$$

where *N* denotes the number of molecules, and *a* represents the fitted slope of the *MSD* curve. The diffusion coefficient can be determined by using the *a* value in place of the differential approximation in Equation (2).

The diffusion coefficients obtained from the fitted slopes of the *MSD* curve are shown in Table 1. The results show that the BN nanoparticles without surface amination treatment in different types exhibited repulsion of water molecules, reflecting the hydrophobic properties of BN materials. Additionally, BN nanoparticles with a larger surface area exhibited stronger hydrophobic properties (see Table S2 in the Supplementary Materials). BN nanoparticles subjected to surface amination treatment showed inhibited motion of water molecules, with the inhibition strength decreasing in the order A-BN_NSphere > A-BN_NTube > A-BN_NSheet. The calculated diffusion coefficient of water molecules in the W model was 0.1467 Å^2^/ps, and it decreased to only 0.0799 Å^2^/ps in A-BN_NSphere, representing a reduction of about 45.54%. Therefore, the results of the above analyses suggest that BN nanoparticles with different types exhibited significant differences in the diffusion behavior of water molecules. BN nanospheres modified by amination exhibited the strongest inhibition effect on the diffusion of water molecules in MO. Centroid trajectories can be used to visualize the movement of selected molecules within the system over time, fully revealing their diffusion range and thus illustrating their diffusion ability. The motion range of the centroid trajectories of the water molecule is consistent with the diffusion coefficient (see Section S2.2 in the Supplementary Materials).

#### 2.1.2. Average Number and Types of Hydrogen Bonds

The number of hydrogen bonds, including those formed in the entire model and those formed between water molecules and BN nanoparticles, was calculated by averaging the MD trajectories of 300 ps for each model, with each trajectory consisting of 600 frames of consecutive configurations. Hydrogen bonds play a key role in the interaction between water molecules and MO. The definition of hydrogen bonds is based on energetic and geometric criteria, with the latter being commonly used in molecular dynamics [35], as shown in Figure S6.

For each output trajectory of the model developed in this work, the number of hydrogen bonds was counted and averaged, including the hydrogen bonds between water molecules and BN nanoparticles. The results of the statistical calculations are presented in Table 2. The A-BN_NSphere model exhibited the highest average number of hydrogen bonds between water molecules and BN nanoparticles, which can significantly influence the interaction between water molecules and MO. This was due to the introduction of -NH_2_ groups in the BN nanomodels subjected to amination treatment. The number of -NH_2_ groups in A-BN_NSphere is significantly higher than in other BN nanoparticles with the same mass fraction. The introduced -NH_2_ groups are more likely to form hydrogen bonds with water molecules in the oil.

**Figure 2:**
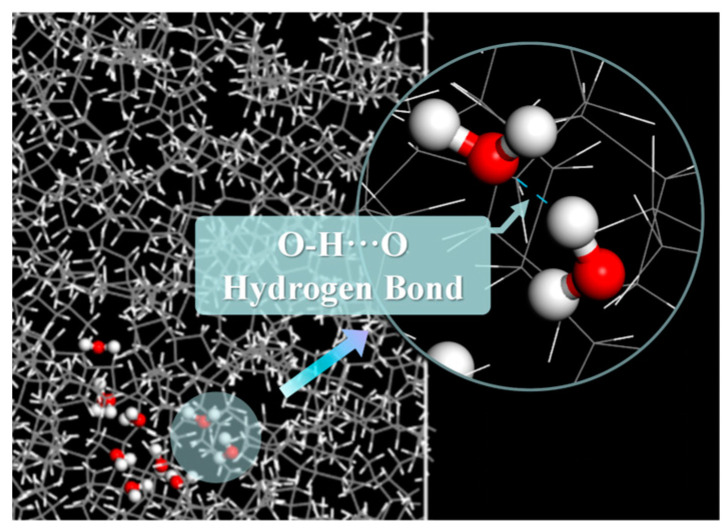
Illustration of a hydrogen bond in the W model. O atoms are shown in red, H atoms in white.

**Figure 3:**
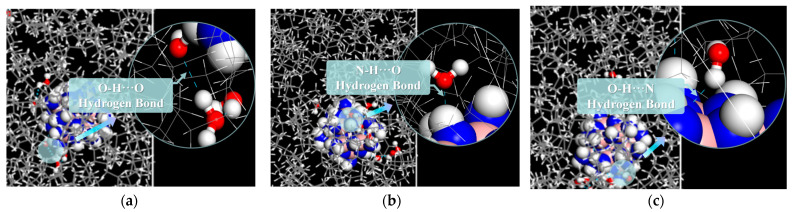
Illustration of hydrogen bonds in the A-BN_NSphere model: (**a**) O–H···O, (**b**) N–H···O, and (**c**) O–H···N hydrogen bonds. O atoms are shown in red, H atoms in white, N atoms in blue, B atoms in pink, and C atoms in grey.

To investigate the internal mechanism in detail, we compared the types of hydrogen bonds formed in the W and A-BN_NSphere models. Figure 2 illustrates the types of hydrogen bonds in the W model, while Figure 3 shows the hydrogen bonds in the A-BN_NSphere model, with blue dashed lines indicating the hydrogen bonds formed between different species.

As shown in Figure 2, only one type of hydrogen bond existed in the W model, specifically the O–H···O hydrogen bond formed between water molecules. Owing to the strong electronegativity of O atoms, water molecules are easily attracted to each other through hydrogen bonding and polymerize to form water molecule clusters in MO. This further leads to a “small bridge” effect in the transformer insulating oil, facilitating thermal breakdown and accelerating the aging of the transformer insulation system.

As shown in Figure 3, three main types of hydrogen bonds were present in the A-BN_NSphere model: (a) O–H···O hydrogen bonds between water molecules, (b) N–H···O hydrogen bonds between the surface of BN nanoparticles and water molecules, and (c) O–H···N hydrogen bonds. The addition of A-BN_NSphere nanoparticles resulted in increased numbers and types of hydrogen bonds in the system. This increase is mainly due to the -NH_2_ groups introduced by the amination of the surface-treated BN nanoparticles. These -NH_2_ groups are highly prone to forming hydrogen bonds with water molecules, consistent with the results discussed in the previous subsection. Additionally, reducing the formation of water molecule clusters in the insulating oil ultimately limits the aging of the internal insulation system of the transformer due to moisture.

#### 2.1.3. Interaction Energies

Despite the fact that the classical force field cannot accurately reflect the true energy value of the system, it is still capable of describing the trend of the interaction energy between classical particles in different classical systems [36,37,38]. Therefore, the interaction energies between water molecules and the background media (MO molecules with or without nanoparticles) in different models were calculated using the Dreiding force field in order to qualitatively investigate the underlying mechanisms of water diffusion behavior in different nano-composited MO models. The interaction energy between two species in the established model can be calculated using Equation (3):

(3) $$E_{inter}=E_{total}-\left(E_{media}+E_{water}\right)$$

**Figure 4:**
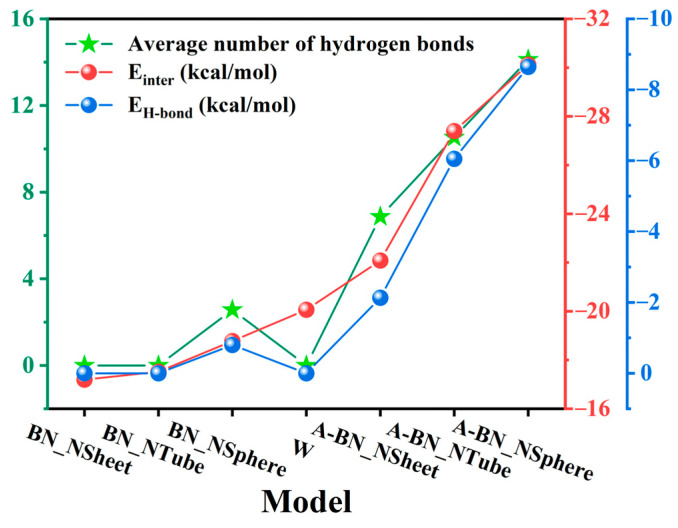
The interaction energy, hydrogen bond energy, and the average number of hydrogen bonds in different types of BN-doped MO models.

In this equation, $E_{inter}$ represents the total interaction energy between water molecules and MO molecules with or without nanoparticles, $E_{total}$ represents the total potential energy of the entire system (water molecules and MO molecules with or without nanoparticles), and $E_{media}$ and $E_{water}$ represent the potential energies of background media molecules (MO molecules with or without nanoparticles) and water molecules, respectively. When $E_{inter}=0$, there is no interaction between the two species, whereas $E_{inter}>0$ and $E_{inter}<0$ indicate repulsive and attractive interactions between them, respectively. The larger the absolute value of $E_{inter}$, the stronger the interaction between the two species [39]. Hydrogen bond (H-bond) energy is also crucial to understanding the diffusion mechanisms of water molecules in various BN-doped MO models. This energy can either be calculated using the ALMO-EDA (Absolutely Localized Molecular Orbital Energy Decomposition Analysis) method based on quantum calculations [40] or by decomposing the total interaction energy calculated by a classical force field according to reference [41]. By integrating scripts into the Materials Studio software 5.5, we decomposed interaction energy into hydrogen bond energy (*E*_H-bond_), van der Waals energy (*E*_vdW_), and electrostatic energy (*E*_electrostatic_) [42]. As shown in Figure 4, the total interaction energy between water molecules and BN-doped MO media (*E*_inter_), the H-bond energy formed between water molecules and BN-doped MO media (*E*_H-bond_), and the number of H-bonds formed between water molecules and BN-doped MO media were calculated.

Figure 4 shows the interaction energy, hydrogen bond energy, and average hydrogen bond numbers in different BN-doped MO models. The absolute values of this interaction energy in the models doped with unmodified BN nanoparticles were smaller than those in the W model. Conversely, the values obtained for the models subjected to surface amination were larger than those of the W model. Moreover, the absolute value of the interaction energy in the A-BN_NSphere model was the largest, 36.40% higher than that of the A-BN_NSheet model; this indicates that the strongest interaction between MO and water molecules was found in the A-BN_NSphere model. The main reason for this effect is that water molecules and -NH_2_ groups introduced on the surface of BN nanoparticles are polar species with a strong mutual attraction. BN_NSphere had the largest number of amino grafting sites on its surface, leading to the highest number of -NH_2_ groups in the A-BN_NSphere model. This contributes to the highest interaction energy between MO and water molecules, leading to a lower diffusion coefficient of the latter and effectively suppressing their diffusion in oil. Moreover, when MO models are doped with untreated BN nanocomposite, the H-bond energy and the number of H-bonds have a minimal contribution, indicating that H-bonds are rarely formed between BN nanoparticles without surface amination. On the other hand, the increase in the absolute value of H-bond energy accounts for the majority of the increase in the absolute value of interactions in A-BN_NSheet, A-BN_NTube, and A-BN_NSphere models, indicating that the H-bonds formed between water molecules and BN-doped MO media are responsible for restricting the diffusion behavior of water molecules in these three models.

#### 2.1.4. Fractional Free Volumes

A hard-ball probe model was used to calculate the free volume of the system based on the Connolly Surfaces of our various atomic models [34,43,44]. We assume that the probe is an impenetrable sphere and moves inside the molecular models shielded by the Connolly Surfaces. As shown in Figure S7, the region that the probe cannot pass through is considered to occupy the volume, while the region that the probe can pass through is considered the free volume. After continuous refinement and application in various studies, fractional free volume (*FFV*) is one of the factors that affect the diffusion ability of molecules in the system. *FFV* represents the ratio of the free to the total volume in the system, as shown in Equation (4):

(4) $$FFV=\frac{V_{f}}{V_{f}+V_{O}}\times 100\%$$

where *V_f_* denotes the free volume, representing the region through which the probe can pass, and *Vo* denotes the occupied volume, representing the region through which the probe cannot pass. The diameter of the probe ball used in our calculation is 1.6 Å. The free volume is distributed in the system in the form of “holes”; when a channel is formed between two holes, small molecules can rapidly jump from one hole to another, thus completing their diffusion in the system. The results averaged over the 300 ps MD trajectory are shown in Table 3, and the single-frame-visualized *FFV* calculation for different models is shown in Figure S8.

The free volume is an important indicator of the diffusion behavior of molecules in different systems. However, it is not the only factor that influences the diffusion behaviors of molecules in different systems. Thus, we have divided our models into two categories: models containing BN doping without surface modifications and those containing BN doping with surface modifications. In the same classification, the order of *FFV* in different models is as follows: BN_NSheet > BN_NTube > BN_NSphere and A-BN_NSheet > A-BN_NTube > A-BN_NSphere, respectively, which is consistent with the diffusion coefficient trend of water molecules. In other words, the greater the *FFV*, the faster the diffusion of water molecules. Moreover, the *FFV* of BN_NSphere is the smallest among all the models.

In those models doped with the same type of BN nanoparticles (e.g., BN_NSheet and A-BN_NSheet), the *FFV* is lower in those models containing BN doping with surface amination treatments than those without surface amination treatments. This result is also consistent with the diffusion coefficient trend of water molecules in different models, indicating that the surface amination of BN nanoparticles reduces the diffusion ability of water molecules.

Overall comparison of these six models shows that *FFV* does not follow the same trend as the diffusion coefficient of water molecules, indicating that *FFV* is not the only factor determining the diffusion behavior of water molecules. The strong attractive interaction energy between amino groups and water molecules on the surface of BN nanoparticles plays a key role in restricting the diffusion of water molecules. In other words, the diffusion behavior of water molecules is mainly dominated by the strong attractive interaction between the amino group and the water molecule on the surface of the nanoparticle and is not only determined by *FFV*.

To provide a comparison, similar simulations were also conducted on three types of hydroxylated-BN-doped MO models (see Section S2.8 in the Supplementary Materials).

### 2.2. Effect of Different Temperatures on the Diffusion of Water Molecules in the A-BN_NSphere Model

The above simulations and analyses showed that the nanosphere is the optimal doping type for BN nanoparticles, effectively hindering the diffusion of water molecules in MO. Additionally, because the temperature significantly influences the insulating properties of transformer oil, we also examined the effect of A-BN_NSphere nanoparticles at different temperatures. Considering that the normal operating temperature of transformers ranges from 30 to 90 °C, we set the simulation temperatures of the A-BN_NSphere model to 303, 323, 343, 363, and 383 K in subsequent calculations to explore the microscopic mechanism under different temperatures.

#### 2.2.1. Diffusion Coefficients at Different Temperatures in the A-BN_NSphere Model

**Figure 5:**
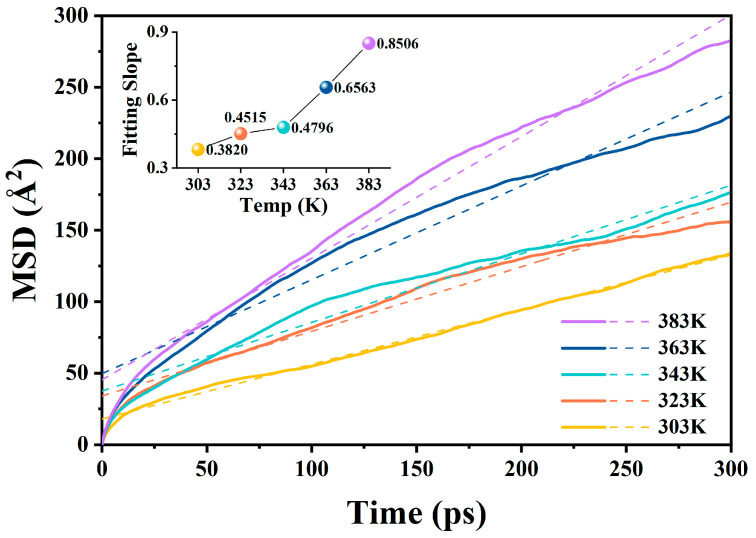
*MSD* curves of water molecules at different temperatures in the A-BN_NSphere model.

The *MSD* curves of water molecules in oil and their diffusion slopes in the A-BN_NSphere model at different temperatures are shown in Figure 5 and Table 4. The temperature significantly affected the diffusion behavior of water molecules in MO. The slope of the *MSD* curve increased with the simulation temperatures, indicating a faster diffusion of water molecules in oil as the temperature increased. This finding is consistent with thermodynamic motion theory: the higher the temperature, the more vigorous the movement of liquid molecules. The collision probability and intensity between molecules in the model increase with increasing temperature; hence, a water molecule experiences a higher number of stronger collisions with other molecules along different directions at the same time, which leads to a more intense diffusion behavior of water molecules in oil.

#### 2.2.2. Hydrogen Bonds and Radial Distribution Functions

The changes in the number of hydrogen bonds calculated for the present model at different temperatures are shown in Table 5. The average number of hydrogen bonds in the model decreased with increasing temperature, both in terms of the total number of hydrogen bonds and the number of hydrogen bonds between water molecules and A-BN_NSphere. To investigate in detail the effect of different temperatures on the hydrogen bonds formed between different atoms, the latter were labeled according to the types of hydrogen bonds present in the A-BN_NSphere model. The radial distribution functions (RDFs) between O and H atoms as well as between N and H atoms were then analyzed. The RDF reveals the characteristics of the spatial distribution of a system and can be used to analyze interatomic interactions and the relative positions, describing the strength and type of intermolecular interactions.

**Figure 6:**
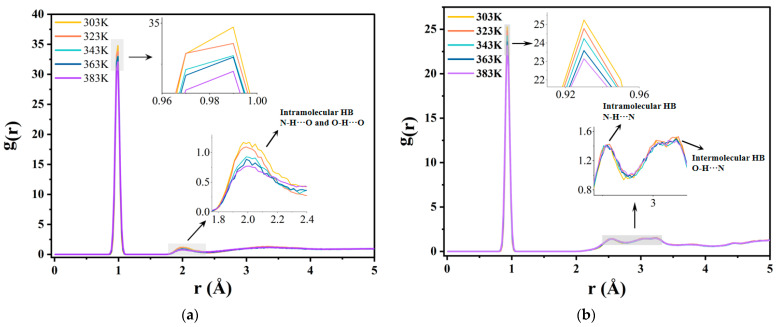
RDFs of O–H and N–H pairs at different temperatures: (**a**) O–H RDF and (**b**) N–H RDF.

At distances exceeding 5 Å, the RDF curve becomes smooth and approaches a stable value; hence, only the data within the first 5 Å were plotted and analyzed in this paper. The specific results are shown in Figure 6. The O–H and N–H RDFs exhibited similar trends. At short distances, all RDF values are 0. This is because the distance from the reference particle is smaller than the minimum distance required for chemical bond formation between atoms. Consequently, no target atoms can be found within the calculated volume of the spherical shells, resulting in values of 0. In the intermediate range, the atoms shift from their original positions due to movements around their equilibrium positions, creating peaks in the RDF curves that characterize atomic stacking and bonding. Finally, the RDF values converge to 1. This is because, at long range, the probability of finding atoms at a given distance is essentially the same, so that the local density matches the overall density, resulting in values of 1. As a result, the *g*(*r*) curve flattens as r increases and eventually converges to the constant value of 1.

Focusing on the intermediate distance range, the peaks that formed near 1 Å in both (a) and (b) plots in Figure 6 originate from the direct chemical bonds formed between atoms, which in this case correspond to the chemical bonds between O and H atoms in water molecules and between N and H atoms in BN nanospheres, respectively. As the temperature increases, the thermal motion of the atoms in these bonds intensifies, leading to a decrease in peak height and an increase in the corresponding width. This suggests that the direct chemical bonds in the model gradually weaken with increasing temperature, becoming more susceptible to breakage.

According to Figure 6a, the H-bonds formed between O and H are primarily those formed between water molecules and between water molecules and amino groups on the surface of BN molecules, which decrease as the temperature increases. In contrast, Figure 6b shows two peaks associated with H-bonds. The first peak around 2.5 Å represents the H-bonds that form between amino groups on the surface of the BN nanospheres (shown in Figure S9 in the Supplementary Materials), while the second peak around 3.2 Å represents the intermolecular hydrogen bonds that form between BN nanoparticles and water molecules (O-H···N hydrogen bonding, as shown in Figure 3).

#### 2.2.3. Temperature Dependence of Interaction Energy

**Figure 7:**
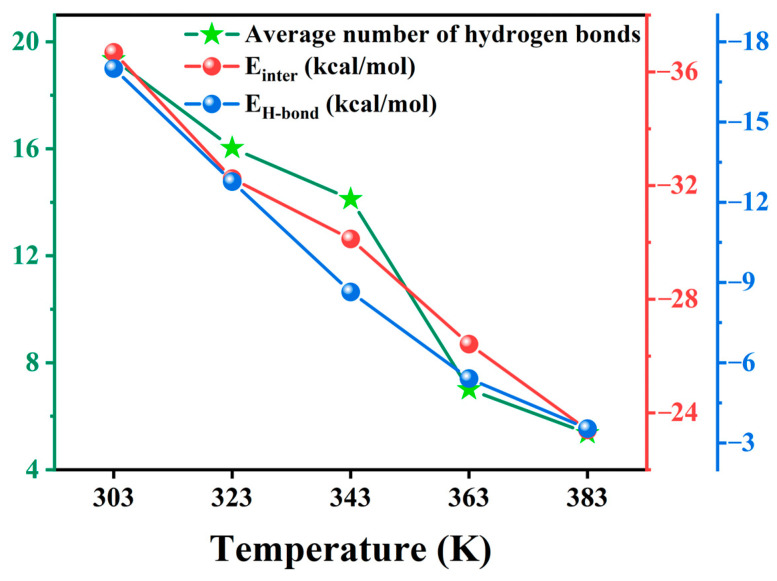
The interaction energy, hydrogen bond energy, and average number of hydrogen bonds in the A-BN_NSphere model under different temperatures.

As shown in Figure 7, the total interaction energy between water molecules and A-BN_NSphere-doped MO media (*E*_inter_), the H-bond energy formed between water molecules and A-BN_NSphere-doped MO media (*E*_H-bond_), and the number of H-bonds formed between water molecules and A-BN_NSphere-doped MO media were calculated. As the temperature increases, the interaction energy, the number of H-bonds, and the H-bond energy between water molecules and A-BN_NSphere-doped MO media decreases, with the decreasing trend of H-bond energy and interaction energy being consistent. Accordingly, the decrease in the absolute value of interaction energy is mainly due to a decrease in the absolute value of H-bond energy. As the temperature increases, the H-bond network weakens, which enhances the diffusion behavior of water molecules in the MO. Additionally, the increased diffusive motion of water in oil can be attributed to the rise in the free volume fraction in the model. The free volume of the model gradually increases with the temperature, as shown in Section S2.7 in the Supplementary Materials. Higher diffusion coefficients of water molecules in oil indicate greater directional or nondirectional displacements of the molecules. The increased diffusion ability of water molecules makes it easier for them to overcome potential barriers imposed by the surrounding environment. This allows them to move and aggregate under the influence of the applied electric field, ultimately resulting in the degradation of the electrical and insulating properties of transformer oil.

### 2.3. Electrical Properties of BN/MO Interface Models

#### 2.3.1. Charge Density Differences

**Figure 8:**
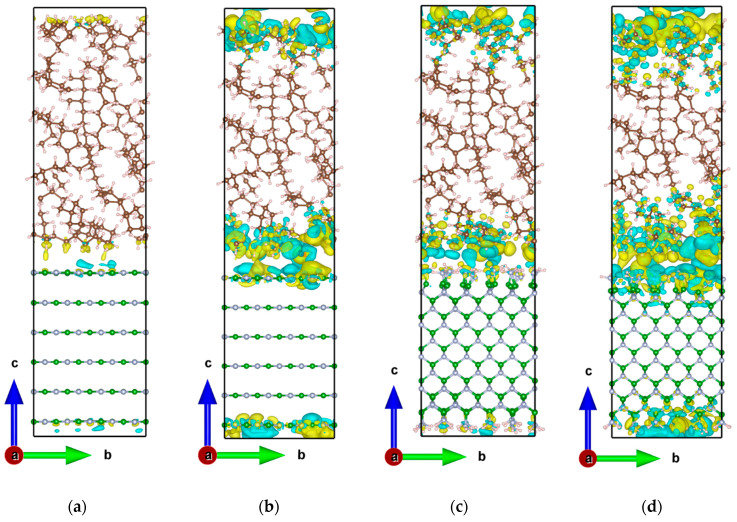
Charge density differences of BN/MO interfacial models with or without the addition of water molecules: (**a**) h-BN/MO, (**b**) h-BN/H_2_O/MO, (**c**) c-BN/MO, and (**d**) c-BN/H_2_O/MO. The isosurface level was uniformly set to 0.0002 e bohr^−3^, with yellow and cyan areas indicating electron accumulation and depletion, respectively. O atoms are shown in red, H atoms in pink, N atoms in blue, B atoms in green, and C atoms in brown.

The charge density difference is one of the important tools for studying the electronic structure, which can intuitively show the charge transfer after the interaction between the interfaces. The charge density was calculated in VASP by solving the Kohn–Sham equations based on DFT, using plane-wave basis expansions and self-consistent field (SCF) iterations. During the calculation, the pseudopotential method is used to simplify the complex electronic interactions [45]. See the Supplementary Materials for the detailed calculation procedure and algorithms. To determine the effect of adding water molecules and amino groups on the charge distribution at the interface, the charge density difference of each interfacial structure was calculated using the following equation:

(5) $$\Delta \rho =\rho_{Total}-(\rho_{BN}+\rho_{MO}+\rho_{H_{2}O})$$

where $\rho_{Total}$ represents the total charge density, while $\rho_{BN}$, $\rho_{MO}$, and $\rho_{H_{2}O}$ are the charge densities of BN, MO, and water molecules, respectively. The results are shown in Figure 8, with yellow and cyan areas indicating electron accumulation and depletion, respectively. To facilitate direct comparisons between different interfaces, the isosurface level was uniformly set to 0.0002 e bohr^−3^, meaning that the surface does not show charge shifts below this value, and only areas above it are visible. h-BN refers to the BN layer generated by the unit cell corresponding to BN nanosheets, so h-BN/MO represents the interface between the h-BN layer and the MO layer. c-BN refers to the BN layer generated by the unit cell corresponding to BN nanospheres, and as a result, c-BN/MO represents the interface model between the c-BN and MO layers. h-BN/H_2_O/MO and c-BN/H_2_O/MO represent the two corresponding interfacial models with water molecules at the interface to investigate their effect on electrical properties such as the interfacial potential barrier.

**Figure 9:**
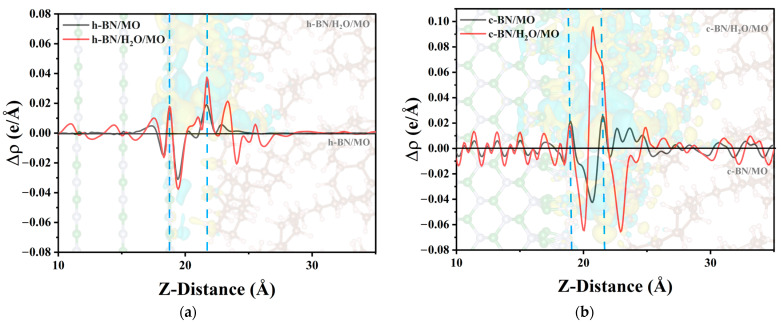
Averaged charge density differences along the Z-direction: (**a**) h-BN/MO and (**b**) c-BN/MO interface models. In between the blue dotted lines is the interfacial area between the BN layer and the MO layer.

Figure 9 shows the total charge density difference along the Z-axis calculated by integration. The area between the two blue dashed lines in the figure indicates the interfacial region between the BN and MO layers. For the h-BN/MO interface model without water molecules, only a slight charge density difference was observed at the interface, indicating minimal interfacial charge transfer between h-BN and MO (Figure 8a). Overall, h-BN loses a small number of electrons at the interface, while the hydrogen atoms of MO gain a small amount. The interfacial charge transfer primarily occurs in the interface region, with minimal impact on the charge of the MO bulk, as shown in Figure 9a. With the addition of water molecules at the interface, the interfacial charge transfer increases and has a greater effect on the charge in the inner region of BN. Moreover, the oxygen atom of the water molecule can easily capture electrons, which results in a smaller positive peak at the interface (20 Å range). The charge transfer at the c-BN/MO interface is significantly larger than that at the h-BN/MO interface, due to the amino groups on the c-BN surface; additionally, some hydrogen atoms in MO lose electrons. The presence of water molecules at the interface accentuates this phenomenon, as shown in Figure 9b. However, the isosurface level was set to a very small value (only 0.0002) in this work, to reflect the weak effect of water molecules. The overall effect of water molecules on the charge transfer is minimal, with a charge density difference at approximately the order of magnitude of 10^−2^.

#### 2.3.2. Local Density of States

**Figure 10:**
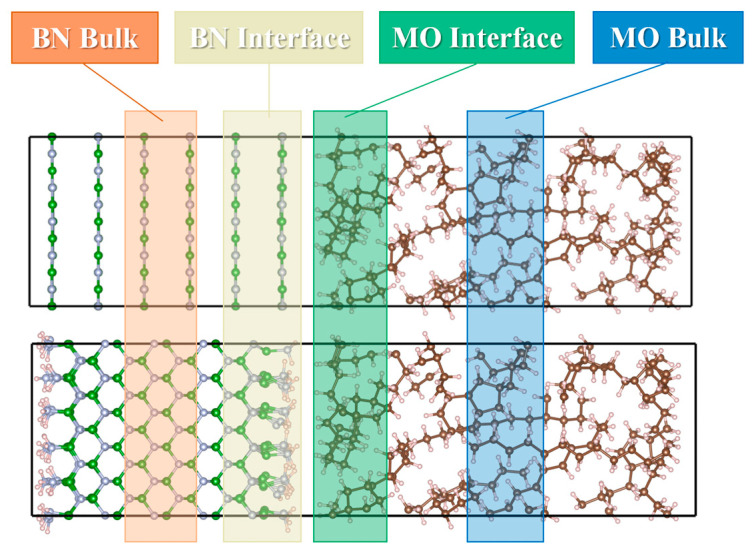
Schematic illustration of the partitioning of the interface model in different regions to investigate LDOS.

To investigate the root cause of the charge transfer behavior discussed above, the electronic density of states (DOS) of the corresponding model was calculated and projected onto BN, MO, and water molecules. However, MO is an insulator, and the above analyses show that the charge transfer mainly occurs in the region near the interface, whereas the local density of states (LDOS) can represent the electronic DOS near a specific location. Therefore, based on the distance relationship at the interface, MO atoms in specific regions were selected for LDOS calculations. These regions were named BN surface, MO surface, BN bulk, and MO bulk, according to their position concerning the interface and the bulk regions, as shown in Figure 10.

**Figure 11:**
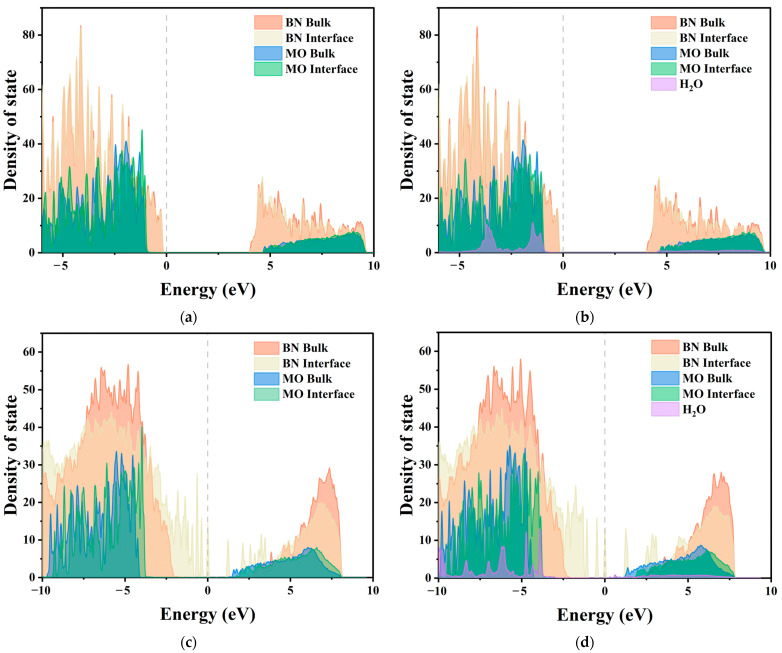
Local and projected DOS of BN/MO interfaces with or without water molecules: (**a**) h-BN/MO, (**b**) h-BN/H_2_O/MO, (**c**) c-BN/MO, and (**d**) c-BN/H_2_O/MO.

The corresponding LDOS curves for the four models, shown in Figure 11, all had a Fermi energy level of 0 eV. Figure 11 shows that doping BN nanoparticles in MO generates electron- or hole-type traps in MO, with trap energy levels near the conduction band bottom or valence band top. Theoretically, contact charge transfer occurs through two main pathways. The first is electron tunneling, which involves electrons directly passing from the valence band of the insulator into the conduction band of the conductor, regardless of the potential barrier at the interface [46]. The second pathway relies on defect states in the band gap of the insulator, allowing electrons to pass through [47,48]. The Fermi energy levels are always located in the band gap rather than the conduction band of MO, suggesting that MO gains electrons through defect states rather than electron tunneling. In contrast, c-BN has amino polar groups on its surface, introducing more traps and defect states compared to h-BN, resulting in a stronger ability to accept or lose electrons. The comparison of Figure 11a,b shows that water molecules do not introduce defect states, mainly affecting the interface region and with a lower impact on the bulk region. In contrast, water molecules in c-BN increase the amount of defect states in the band gap, enhancing the electron transfer. These findings are in agreement with the results of the charge difference density analysis.

#### 2.3.3. Potential Distributions and Barriers across the BN/MO Interface

**Figure 12:**
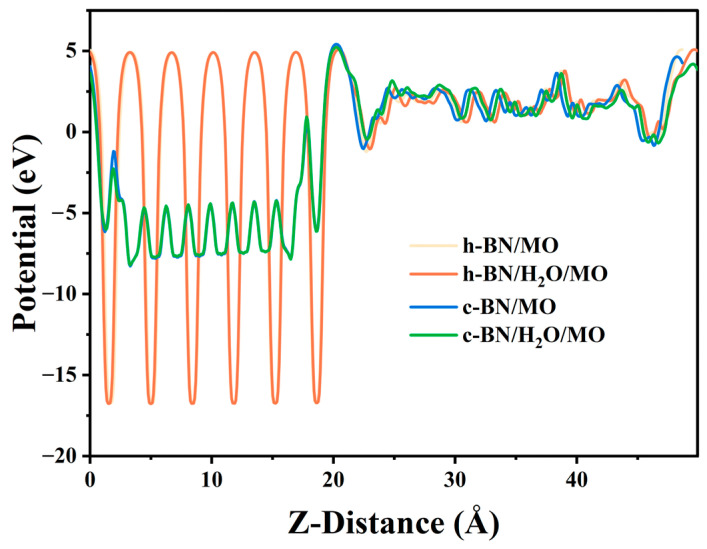
Potential distribution along the Z-axis of four BN/MO interfacial models.

To calculate the interfacial potential barrier between BN and MO and the effect of water molecules on it, we first calculated the potential distributions of the four models using the PBE GGA functional. Using the energy of the MO bulk region, which is the same for all four models because it is not affected by the interfacial charge transport [49], the h-BN/MO model was used as a reference to align the potentials, and the corresponding results are shown in Table 6. Table 6 shows that the presence of water molecules at the interface reduced the potentials of h-BN/H_2_O/MO and c-BN/H_2_O/MO to only 0.0857 and 0.0998 eV, respectively. The aligned potential distribution results are shown in Figure 12. Figure 12 shows that the interfacial potential distributions of BN models with different structures varied significantly. To quantitatively analyze the potential variations in different models, the average potential values in the ranges of 6.6840–13.5417 and 32.0779–37.7719 Å were used to approximate the potentials of the BN and MO layers, respectively. The average potential energy of the BN layer is shown in Table 7.

Furthermore, because changes in the valence band maximum (VBM) and conduction band minimum (CBM) positions affect the interfacial potential barrier between BN and MO, the more accurate PBE0 hybrid functional was used to calculate the energy band structure in this work, thereby avoiding underestimating the band gap value (*E_g_*) [50,51]. However, as the PBE0 functional is too computationally demanding for a large interfacial structure such as the present one, the band alignment method was used to calculate the potential barriers at the BN/MO interface [52,53]. The atomic structures of h-BN, c-BN, and MO were reconstructed using lattice parameters of 2.50 Å × 4.31 Å × 6.65 Å for h-BN, 3.59 Å × 3.59 Å × 3.59 Å for c-BN, and 15.13 Å ×15.13 Å × 15.13 Å for MO. The k-point sampling was 13 × 8 × 5 for h-BN, 9 × 9 × 9 for c-BN, and gamma point only for MO. Then, the average potential, VBM, CBM, and *E_g_* values were calculated using the PBE0 hybrid functional, as shown in Table 8.

**Figure 13:**
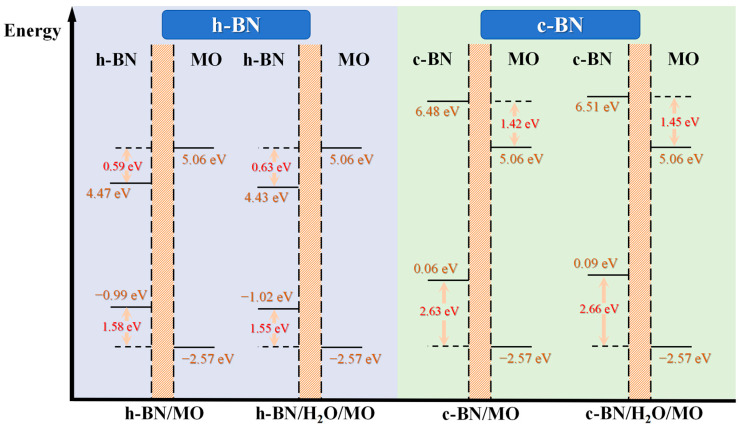
Potential barriers at BN/MO interfaces.

The band gap of MO (~7.63 eV) is 2.2 eV larger than the value calculated using the PBE functional, indicating that the latter significantly underestimates the *E_g_* value. Finally, the average potential energy values of MO and BN in their interfacial structures (Table 6 and Table 7) were used as reference energies to align the electronic structures of BN and MO (Table 8) and calculate the interfacial potential barrier distributions, as shown in Figure 13.

Significant differences were observed among the specific effects of different BN structures on the electronic behavior at the interface. The different roles of the two materials in the electron transport process can be revealed by comparing the energy level diagrams of h-BN and c-BN in Figure 13. In the region of the CBM, h-BN nanoparticles in MO act as effective electron traps. Conversely, c-BN materials act as electron potential barriers, hindering the electron flow. The inspection of the region of the VBM revealed that both h-BN and c-BN can act as hole traps, i.e., they are able to effectively trap holes and thus regulate their transport. Notably, the introduction of water molecule impurities at the interface has a weak effect on the interfacial potential barrier. This observation agrees with the findings in Figure 8. Deeper traps can immobilize and stabilize electrons more effectively, preventing them from moving freely. Additionally, a higher potential barrier acts as a physical obstacle to electron flow, further restricting electron migration through the insulating layer. These properties significantly enhance the electronic coupling between insulating oil and nanoparticles. This improved electronic coupling enhances the overall stability of the insulation system, reduces the risk of partial discharges and breakdowns, and significantly boosts the insulation performance of the transformer.

## 3. Molecular Modeling and Simulation Details

### 3.1. Molecular Dynamics Simulations of Diffusion Behavior of Water Molecules in MO/BN Composite Models

**Figure 14:**
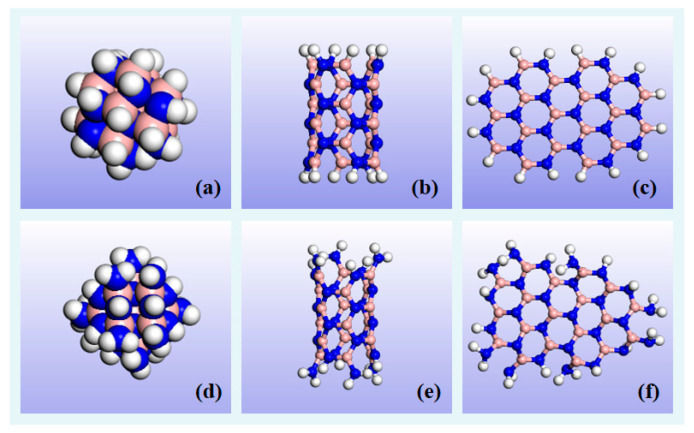
Models of BN nanoparticle with or without surface modification: (**a**) BN_NSphere, (**b**) BN_NTube, (**c**) BN_Nsheet, (**d**) A-BN_NSphere, (**e**) A-BN_NTube, and (**f**) A-BN_NSheet. H atoms in white, N atoms in blue, B atoms in pink.

The MO model was established using Karamay 25# insulating oil, with alkanes and cycloalkanes accounting for 88.6% of all compounds that are commonly used as liquid insulating media in transformer insulation systems [54]. In the modeling process, typical chain alkane and cycloalkane molecules that fully reflect their physical and chemical properties were selected as the component molecules of this type of MO. The corresponding parameters, such as components and mass fractions, are shown in Table S1 and Figure S1. According to references [19,24,37], although nanoparticle diameters can exceed tens of nanometers, diameters no smaller than 10 Å are commonly used to model their molecular structure due to their size effect on water diffusion behavior being smaller than their surface functional groups. Therefore, three different types of BN nanoparticle models were built: BN nanospheres (BN_NSphere) with a diameter of 10 Å, BN nanotubes (BN_NTube), and BN nanosheets (BN_NSheet) with a side length and height of about 18 Å. To ensure that the nanoparticle models were reasonable and stable, unsaturated bonds were saturated with hydrogen atoms, as illustrated in Figure 14a–c. Figure 14d–f show the three BN nanoparticle models subjected to surface amination treatment, named, respectively, A-BN_NSphere, A-BN_NTube, and A-BN_NSheet.

Based on the proportions of alkanes in MO, amorphous structures were modeled using the Amorphous Cell module of the MS software 5.5. Moreover, water molecules with a mass fraction of approximately 1.5 wt.% were added to simulate the insulating oil in a water-containing environment [16,55], resulting in a final model density of 0.84 g/cm^3^, consistent with the experimental value [56]. Three types of models were established and compared in this study: (1) a MO model without BN nanoparticle doping (W), (2) BN nanoparticle models without surface modification (BN_NSphere, BN_NTube, and BN_NSheet), and (3) models of BN nanoparticles subjected to surface amination (A-BN_NSphere, A-BN_NTube, and A-BN_NSheet), for a total of seven models. As an example, the size of the optimized and relaxed BN_NShere composite model was 33.6 Å × 33.6 Å × 33.6 Å. A series of optimization, relaxation, and MD simulation procedures were carried out on the obtained amorphous model using the Forcite module of the MS software 5.5 (see Section S1.1 in the Supplementary Materials). The Dreiding force field, which accurately predicts the properties of BN materials at the molecular level, was used in all simulations [36].

### 3.2. DFT Calculations of Electrical Properties of MO/BN Interface Models

Because the average size of the BN nanoparticles used in the experiments is 130 nm [57], completing high-accuracy molecular-level theoretical calculations with the current computational resources is challenging. Moreover, larger nanoparticles have a smaller surface curvature, making their local structure approximately planar. Therefore, we used the Build Layer module of MS to establish a planar model of the BN/MO interface and study its electrical properties, such as interfacial barriers.

**Figure 15:**
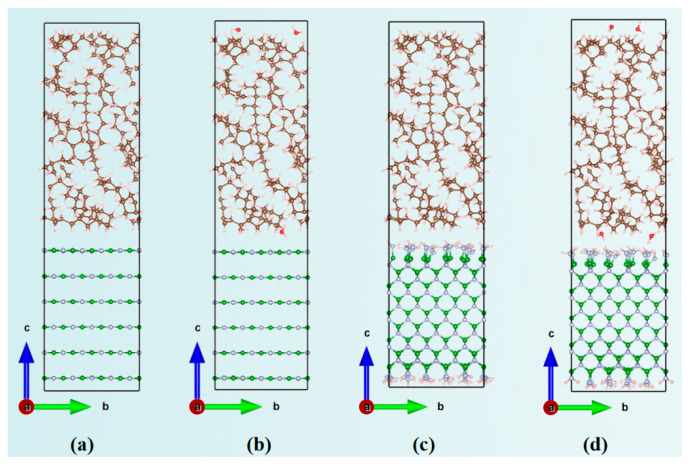
MO/BN interface models: (**a**) model of the h-BN/MO interface; (**b**) h-BN/MO interfacial model with two water molecules inserted at each surface (denoted as h-BN/H_2_O/MO); (**c**) model of the c-BN/MO interface; (**d**) c-BN/MO interfacial model with two water molecules inserted at each surface (denoted as c-BN/H_2_O/MO). O atoms are shown in red, H atoms in pink, N atoms in blue, B atoms in green, and C atoms in brown.

BN nanoparticles can be modeled as two forms on a local scale: a hierarchical sheet structure representing nanosheets and nanotubes, and a cubic structure representing nanospheres. Therefore, two types of BN/MO interfaces, the h-BN/MO interface and the c-BN/MO interface, were modeled using the “Build Layer” tools in Materials Studio. h-BN refers to the BN layer generated by the unit cell corresponding to BN nanosheets, so h-BN/MO represents the interface between the h-BN layer and the MO layer. c-BN refers to the BN layer generated by the unit cell corresponding to BN nanospheres, and as a result, c-BN/MO represents the interface model between the c-BN and MO layers. In order to study the water molecule influence on the interfacial barrier across the BN/MO interface, two water molecules were inserted at the interfaces. h-BN/H_2_O/MO and c-BN/H_2_O/MO represent the two corresponding interfacial models with water molecules at the interface to investigate their effect on electrical properties such as the interfacial potential barrier. For a detailed account of the modeling procedures, see Section S1.2 in the Supplementary Materials. The optimized structures of the MO/BN interface shown in Figure 15 were visualized using the Visualization for Electronic and Structural Analysis (VESTA) software 3.5.8. Then, the initial MO/BN interface models were imported into the VASP software 6.3.1 for structural optimization and quantum calculations based on DFT [58,59,60]. For the exact DFT calculation method and the parameter settings, see Section S1.3 in the Supplementary Materials.

## 4. Conclusions

MD and DFT were employed in this study to investigate how different types and surface conditions of doped BN nanoparticles may affect the dielectric and electrical properties of insulating oil. We investigated the significant differences in diffusion behavior between amination-modified and unmodified BN surfaces, particularly in terms of the potential barrier across the BN/MO interface and the diffusion behavior of water molecules in the model. Accordingly, the following conclusions can be drawn: 

(1) It was found that the amination-modified BN-nanosphere-doped MO model (A-BN_NSphere) has the lowest diffusion coefficient of water molecules. In A-BN_NSphere models, amino grafting sites introduce more -NH_2_ polar groups, which results in numerous hydrogen bonds of various types being formed with water molecules. This results in a stronger interaction between water molecules and MO, a reduction in the free volume fraction, a substantial decrease in the diffusion of water molecules, and an improvement in MO’s insulating properties.

(2) In all our BN-doped MO models, there is a positive correlation between the temperature and the diffusion coefficient of water molecules. With increasing temperature, the intramolecular chemical and hydrogen bonds in the model gradually weaken, and the free volume fraction increases. This facilitates the movement of water molecules, enabling them to overcome potential barriers posed by the surrounding environment more readily. However, the temperature has relatively little impact on the hydrogen bonds formed with BN nanoparticles.

(3) The doping of BN nanoparticles into MO can result in electron traps. In comparison with h-BN, c-BN surfaces contain more amino polar groups that introduce more traps and defect states. In addition, the presence of water molecules at the c-BN interface increases the number of traps in the band gap.

(4) Different types of BN nanoparticles play different roles in electron transport in MO due to the differences in their surface morphology and polar groups. In MO, the h-BN surface (derived from BN nanosheets) acts as a trap for electrons with a trap depth of −0.59 eV, while the c-BN surface (derived from BN nanospheres) acts as a potential barrier for electrons with a potential height of 1.45 eV. Additionally, the presence of water molecules at the interface between BN and MO has little impact on the potential barriers.

The findings of this study reveal the impact of different types of nanoparticles on the insulating properties as well as the electronic behavior of insulating materials. The results of this study may serve as theoretical references for nanomodification technologies aimed at improving the electrical and insulating properties of insulating materials for power equipment.

## Figures and Tables

**Table 1:** Fitted slopes of *MSD* curves and diffusion coefficients of water molecules.

Model	BN_NSheet	BN_NTube	BN_NSphere	W	A-BN_NSheet	A-BN_NTube	A-BN_NSphere
*a*	1.1000	1.0723	1.0493	0.8799	0.7442	0.5752	0.4796
*D* (Å^2^/ps)	0.1833	0.1787	0.1749	0.1467	0.1240	0.0959	0.0799

**Table 2:** Average number of hydrogen bonds.

Model	BN_NSheet	BN_NTube	BN_NSphere	W	A-BN_NSheet	A-BN_NTube	A-BN_NSphere
* HB_Total_	16.1531	16.3117	17.2504	18.4509	19.1165	24.5957	30.0948
* HB_W-BN_	0	0	2.5674	0	6.8686	10.5308	14.1198

* HB_Total_ denotes the total average number of hydrogen bonds; HB_W-BN_ represents the average number of hydrogen bonds between water molecules and BN.

**Table 3:** *FFV* values in different models.

Classification	Model	*V_f_*	*V_O_*	*FFV*
Models without BN surface amination	BN_NSheet	15,434.6428	31,525.2772	32.868%
	BN_NTube	14,728.8692	31,595.7208	31.876%
	BN_NSphere	14,749.7695	31,522.2005	31.795%
Models with BN surface amination	A-BN_NSheet	15,211.6088	31,072.4812	32.866%
	A-BN_NTube	14,342.2594	31,750.8506	31.116%
	A-BN_NSphere	14,035.2070	31,837.9530	30.596%

**Table 4:** Fitting slopes of *MSD* curves and diffusion coefficients of water molecules at different temperatures in the A-BN_NSphere model.

Temperature	303 K	323 K	343 K	363 K	383 K
*a*	0.38200	0.45153	0.47957	0.65631	0.85056
*D* (Å^2^/ps)	0.06367	0.07526	0.07993	0.10939	0.14176

**Table 5:** The average number of hydrogen bonds at different temperatures.

Temperature	303 K	323 K	343 K	363 K	383 K
HB_Total_	33.7608	30.8239	30.0948	26.7774	23.8970
HB_W-BN_	19.3467	16.0300	14.1198	7.0067	5.3667

**Table 6:** Potential shifts of MO in the bulk region.

Model	Average Potential (eV)	Potential Shift (eV)
h-BN/MO	1.7621	0
h-BN/H_2_O/MO	1.6764	−0.0857
c-BN/MO	2.7475	0.9854
c-BN/H_2_O/MO	2.6477	0.8856

**Table 7:** The average potential energy of BN in the bulk region.

Model	h-BN/MO	h-BN/H_2_O/MO	c-BN/MO	c-BN/H_2_O/MO
Average potential (eV)	−2.3935	−2.4274	−6.4960	−6.4638

**Table 8:** Average potential energy of BN in the bulk region.

Structure	VBM (eV)	CBM (eV)	*E_g_* (eV)	Average Potential (eV)
h-BN	1.4057	6.8614	5.4557	0
c-BN	6.5584	12.9735	6.4151	0
MO	−4.3345	3.3006	7.6351	0

## Data Availability

All the data are available within the manuscript. Additional data will be provided upon request from the corresponding authors.

## Supplementary Materials

The following supporting information can be downloaded at: https://www.mdpi.com/article/10.3390/molecules29184500/s1. Figure S1: Atomic structures of MO. Figure S2: Modeling, simulation, and analysis processes of MO/BN composite models. Figure S3: Models and analysis process of BN/MO interfacial models. Figure S4: CHGCAR file. Figure S5: Illustration of centroid trajectories. Figure S6: Geometric criterion for hydrogen bond formation. Figure S7: Schematic diagram of hard-ball probe model. Figure S8: Visualized *FFV* calculation results in different types of BN-doped MO models. Figure S9: The hydrogen bonding between the grafted amino groups on the surface of the BN nanospheres (N-H···N hydrogen bonding). Figure S10: Correlation diagram of *FFV* data at different temperatures. Figure S11: Visualized *FFV* calculation results in different temperatures (in A-BN_NSphere model). Figure S12: *MSD* curves of water molecules in the hydroxylation models. Table S1: Main components and mass fractions of MO. Table S2: Surface areas of BN nanomodels without surface amination treatment in different models. Table S3: Maximum spanned distances of projected point in the plane of projection. Table S4: Fitting slopes of *MSD* curves and diffusion coefficients of water molecules in the hydroxylation models. Table S5: Interaction energy between water molecules and MO in the hydroxylation models. References [61,62,63,64,65,66,67,68] are cited in the Supplementary Materials.
