# Supplementary material for: Molecular Simulation of the Water Diffusion Behavior and Electronic Properties of Boron-Nitride-Composited Mineral Oil
Source: Molecules. 2024 Sep 22;29(18):4500. doi: 10.3390/molecules29184500 (PMC11434289; doi:10.3390/molecules29184500)
Supplement: Supplementary file 1 [file molecules-29-04500-s001.zip › molecules-3197062-supplementary.pdf]

Supplementary Materials: Molecular Simulation of the Water Diffusion Behavior and Electronic Properties of Boron Nitride-Composited Mineral Oil

Yang Wang [1,2,*], Wenchao Yan [1,2], Kunqi Cui [1,2], Chuanhui Cheng[3,*], Yuanyang Ren [4], Kai Wu[4]

1 School of Electronics and Information, Xi'an Polytechnic University, Xi'an 710048, China

2 Xi'an Key Laboratory of Interconnected Sensing and Intelligent Diagnosis for Electrical Equipment, Xi'an Polytechnic University, Xi'an 710048, China

3 Electric Power Research Institute, China Southern Power Grid, Guangzhou 510663, China

4 State Key Laboratory of Electrical Insulation and Power Equipment, Xi'an Jiaotong University, Xi'an 710049, China

*Correspondence:

Yang Wang, Email: wangyang@xpu.edu.cn (Y.W.)
Chuanhui Cheng, Email: chengchui1239@163.com (C. C.)

S1. Algorithms, Modeling and Simulation Details

S1.1. MO/BN Composite Models and Simulation Details

Table S1. Main components and mass fractions of MO.

	Paraffin	Cycloalkane			
		Monocyclic alkane	Bicyclic alkane	Tricyclic alkane	Tetranuclear alkane
Molecular formula	$C_{12}H_{26}$	$C_{14}H_{28}$	$C_{13}H_{24}$	$C_{16}H_{28}$	$C_{16}H_{26}$
Relative molecular mass	170.3	196.4	180.3	220.4	218.4
Mass fraction (wt.%)	11.6	15.5	28.5	23.3	9.7

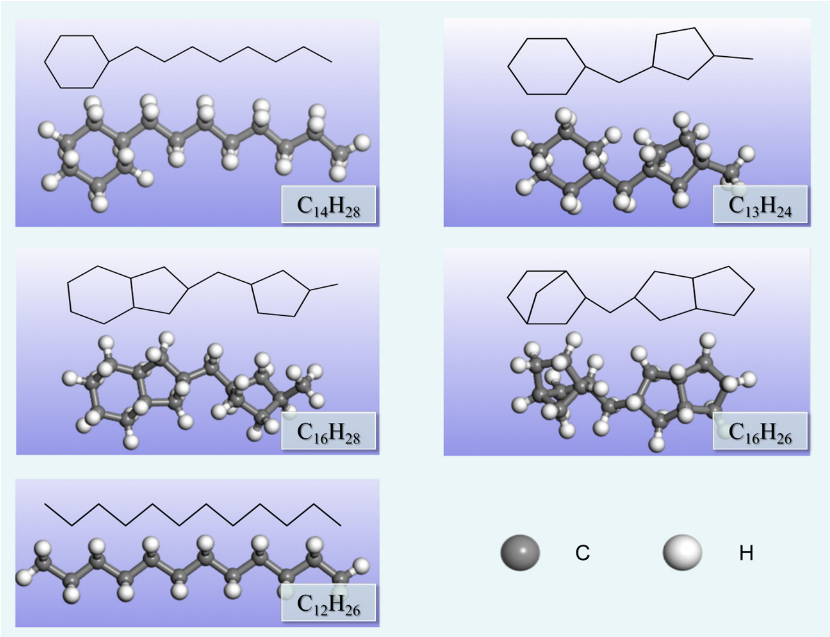

Figure S1. Atomic structures of MO. Grey and white balls represent carbon and hydrogen atoms, respectively.

First, a geometry optimization with an energy convergence value of 10^{-4} kcal/mol (Smart algorithm) was performed on the initial model. Second, five annealing optimization cycles were performed to find the most stable (lowest-energy) structure of the model, with an initial annealing temperature of 300 K and the temperature of each annealing cycle set to 500 K. The model with the lowest energy after annealing was selected for structural relaxation in the NPT ensemble (constant number of particles *N*, pressure *P*, and temperature *T*), transforming the system from the initial nonequilibrium to a relative equilibrium state. Finally, the resulting model was subjected to an MD simulation in the NVT ensemble (constant number of particles *N*, volume *V*, and temperature *T*). During the MD simulation, a Nosé-Hoover thermostat and a Berendsen barostat were used to maintain the temperature and pressure constant, respectively, with the integration step set to 1.0 fs [61]. Taking into account the temperature of the oil-immersed transformer during the actual operation, the simulation temperature and pressure were maintained at 343 K and 0.0001 GPa, respectively [62]. The calculated data were collected every 500 steps during the simulation and used for subsequent analysis. The specific simulation process is shown in Figure S2.

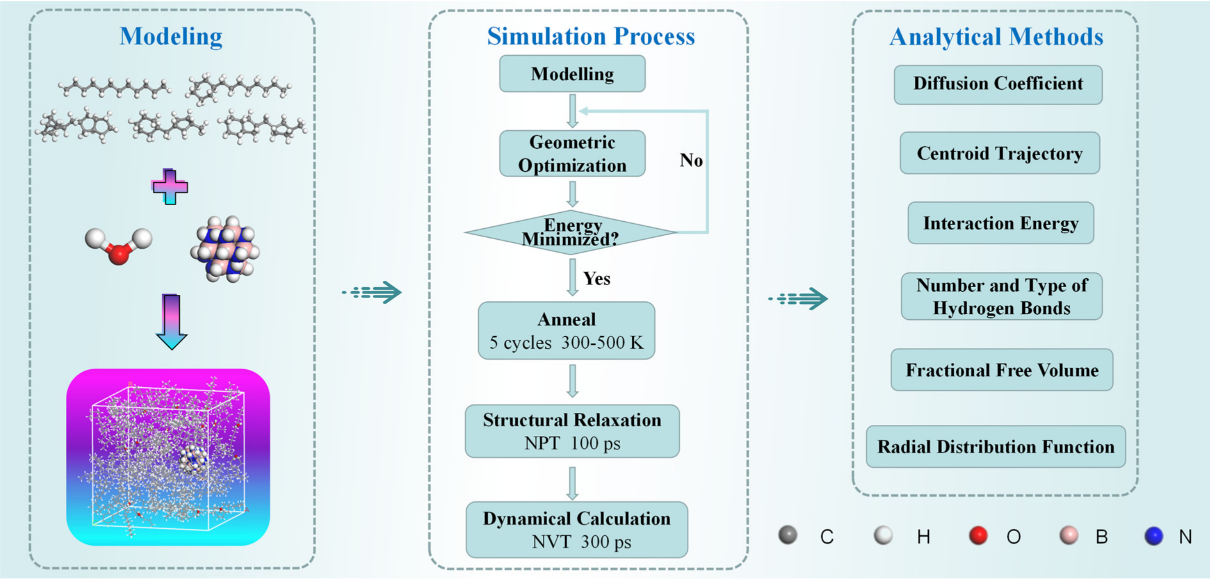

Figure S2. Modeling, simulation, and analysis processes of MO/BN composite models.

S1.2. The Detailed Modelling Procedures of MO/BN Interface Models

The layered sheet structure representing BN nanosheet and nanotube, was derived from the h-BN cell through a 3 × 5 × 4 cell expansion, resulting in lattice parameters in the *ab* plane of 13.04 Å × 12.55 Å. Additionally, the layered sheet structure representing BN nanosphere, was derived from the c-BN cell by faceting and expansion transformations. The BN cell was sectioned along the (1 0 0) plane and subjected to a 5 × 5 × 6 cell expansion, with unsaturated dangling bonds saturated with amino groups. The lattice parameters of the BN cubic supercell in the *ab* plane were 12.78 Å × 12.78 Å.

The initial structure of the amorphous MO layer was built using the Confined Layer tool of the Amorphous Cell module, which helps to reduce the vacuum layer region at the interface. To ensure matching lattice parameters between the BN and MO layers, the cell lengths in the a and b directions of the MO molecular model were controlled to match the ab plane lattice parameters described above, and the density of the model was adjusted by varying the length in the c direction. The BN layer was combined with the MO layer using the "Build Layers" tool to form the initial MO/BN interface model. These models were then optimized using the Dreiding force field, resulting in final lattice parameters of 13.04 Å × 12.55 Å × 50.01 Å for the h-BN interface model and 12.78 Å × 12.78 Å × 50.60 Å for the c-BN interface model. And two water molecules were separately added at the interface to investigate their effect on electrical properties such as the interfacial potential barrier.

S1.3. DFT calculation method of MO/BN Interface Models

The projector-augmented wave (PAW) method was used to describe the interaction between electrons and nuclei [63]. Electron exchange–correlation interactions were described using the Perdew–Burke–Ernzerhof (PBE) generalized gradient approximation (GGA) functional along with D3 van der Waals corrections [64,65]. The GGA–PBE method is highly effective for surface simulations [66]. In this study, $2s^22p^1$ electrons of B atoms, $2s^22p^3$ electrons of N atoms, $2s^22p^2$ electrons of C atoms, and $2s^22p^4$ electrons of O atoms were treated as valence electron shells [58,67]. The plane-wave cut-off energy was set to 550 eV in all calculations, and the energy convergence criterion was set to 1×10^{-5} eV. Moreover, a 3 × 3 × 1 k-point sampling was used in the calculations [68]. The atomic structure was relaxed until the maximum ionic force on each atom was less than 0.02 eV/Å. A schematic procedure of modelling and analyzing interfacial structures of BN/MO is shown in Figure S3.

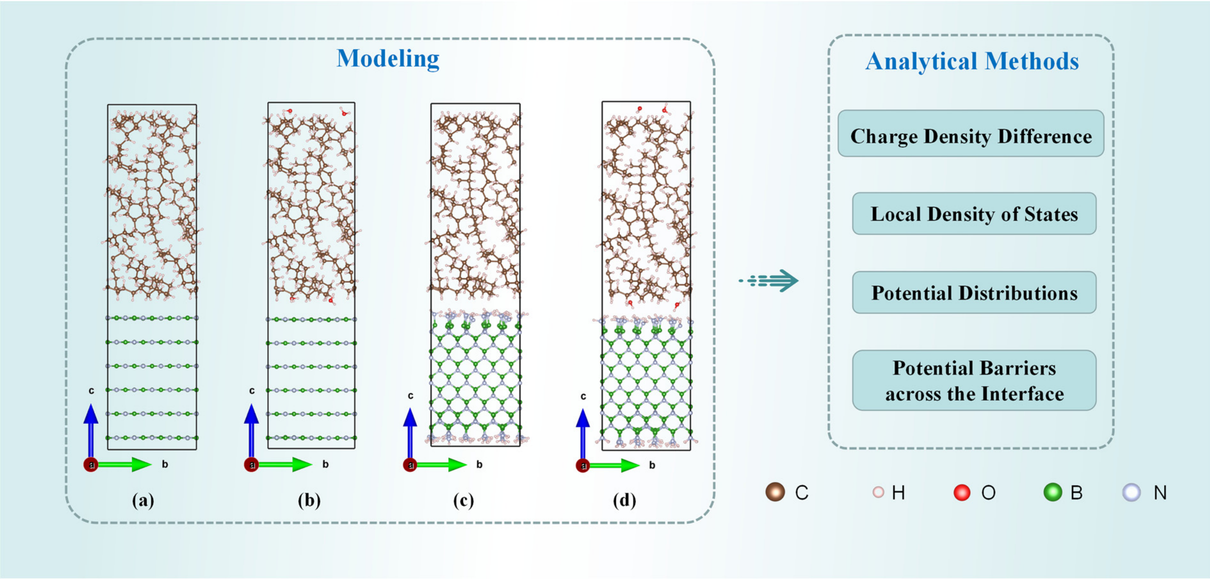

Figure S3. Models and analysis process of BN/MO interfacial models: (a) model of h-BN/MO interface; (b) h-BN/MO interfacial model with two water molecules inserted at each surface (denoted as h-BN/H_2O/MO); (c) model of c-BN/MO interface; (d) c-BN/MO interfacial model with two water molecules inserted at each surface (denoted as c-BN/H_2O/MO).

S1.4. Charge density calculation formula and steps

The charge density was calculated using VASP by solving the Kohn-Sham equations based on DFT, using plane wave expansions and self-consistent field (SCF) iterations [68]. The Kohn-Sham equations are discretized and the wave functions are expanded in reciprocal space and then transformed from reciprocal space back to real space by Fast Fourier Transform (FFT) to obtain the charge density. During the calculation, the pseudopotential method is used to simplify the complex electronic interactions.

The charge density calculation result is stored in CHGCAR file output by VASP. The calculation steps are as follows:

(1). Calculation steps: In VASP, we can get CHGCAR file by static calculation. Set to "NSW=0 and LCHARG=.TRUE." in the INCAR file, these parameters instruct VASP to perform static calculations and save charge density data. The charge density distribution is obtained from the CHGCAR file, which contains the valence electron charge density calculated based on the pseudopotential, and is a lattice file that provides detailed information about the electron density.

(2). The CHGCAR consists of the following blocks:

(2.1). Structure in POSCAR format

(2.2). FFT-grid dimensions NGXF, NGYF, NGZF

(2.3). Charge times FFT-grid volume is written with multiple real numbers per line until all NGXF*NGYF*NGZF values of the block are written.

(2.4). Augmentation occupancies

The CHGCAR file is shown in the following Figure S4. Line 846 of the output file provides the grid numbers in the x, y, and z directions, corresponding to NGXF, NGYF, and NGZF, respectively. The charge density information begins at line 847, storing the charge density values in the order of the FFT grid points.

```
 0.870675  0.738892  0.880771
 0.897896  0.561269  0.907628
 0.766778  0.566188  0.915675
 0.797642  0.751420  0.936781
 0.929256  0.733776  0.930391
 0.928246  0.568070  0.960588
 0.796575  0.590046  0.967592
 0.889622  0.683662  0.978821

 160  144  560
0.65375459585E+02 0.65565615929E+02 0.67173683391E+02 0.70271348662E+02 0.74903886271E+02
0.81073585819E+02 0.88721175581E+02 0.97710741898E+02 0.10782227989E+03 0.11875387446E+03
0.13013361491E+03 0.14153977379E+03 0.15252666838E+03 0.16265356920E+03 0.17151494150E+03
0.17877079742E+03 0.18417475580E+03 0.18759536222E+03 0.18902575799E+03 0.18857945875E+03
0.18647457553E+03 0.18301210369E+03 0.17855361607E+03 0.17350049233E+03 0.16827361084E+03
0.16329159336E+03 0.15894718489E+03 0.15558349854E+03 0.15347327224E+03 0.15280447201E+03
0.15367433397E+03 0.15609140913E+03 0.15998262727E+03 0.16520192424E+03 0.17153967142E+03
0.17873658443E+03 0.18650844012E+03 0.19458525835E+03 0.20275992373E+03 0.21093092316E+03
0.21912030059E+03 0.22745720133E+03 0.23613605967E+03 0.24537318430E+03 0.25538280501E+03
0.26637285044E+03 0.27853695596E+03 0.29201243762E+03 0.30679262419E+03 0.32261518424E+03
0.33887382653E+03 0.35460243260E+03 0.36855835345E+03 0.37939861897E+03 0.38591355078E+03
0.38726397789E+03 0.38316290960E+03 0.37395203941E+03 0.36054903967E+03 0.34427800883E+03
```

Figure S4. CHGCAR file

(3). The charge density is calculated as follows:

$$
\begin{aligned}
&\mathrm{n}(r) = data(r) / (V_{grid} * V_{cell}) \\
&V_{grid} = N_{GXF} * N_{GYF} * N_{GZF} \\
&V_{cell} = |a \cdot (b \times c)|
\end{aligned} \tag{1}
$$

Where V_{grid} and V_{cell} denote the grid volume and the cell volume, respectively; $\mathrm{n}(r)$ is the charge density in units 1/Å^3; N_{GXF}, N_{GYF} and N_{GZF} are FFT-grid dimensions.

The integral of $\mathrm{n}(r)$ over the unit cell yields the number of valence electrons (NELECT):

$$
\mathrm{NELECT} = \int_{V_{cell}} n(r) d^3 r = \sum_{N_X, N_Y, N_Z} data(N_X, N_Y, N_Z) / (N_{GXF} * N_{GYF} * N_{GZF}) \tag{2}
$$

S2. Results and Discussion

S2.1. Surface Area

Surface areas of BN nanomodels without surface amination treatment in different models was calculated and shown in Table S2. BN_NSheet had the largest surface area (367.10 Å^2), followed by BN_NTube (272.04 Å^2), with a difference of 95.06 Å^2. However, BN_NSheet contained more hydrogen atoms on its surface, weakening its repulsion of water molecules and reducing the diffusion coefficient difference between BN_NSheet and BN_NTube. In contrast, BN_NSphere had the smallest surface area (267.30 Å^2) and a certain number of hydrogen atoms on its surface. Therefore, BN_NSphere has the least repulsive effect on water molecules.

Table S2. Surface areas of BN nanomodels without surface amination treatment in different models.

Model	BN_NSheet	BN_NTube	BN_NSphere
**Surface area (Å^2)**	367.10	272.04	267.30

S2.2. Centroid Trajectories of Water Molecules

In this work, the centroid trajectories (X, Y, Z) of all water molecules in the W and surface amination models were calculated at each time step. Then, centroid trajectories were schematically drawn based on these coordinates, intuitively illustrating the diffusion tendency of water molecules in the system.

Figure S5 shows the centroid trajectories of the four models, with red balls indicating the spatial position of the centroid of all water molecules at each time. To facilitate the visualization and comparison of their diffusion ranges, and to avoid visual errors from a single perspective, the figures also show the projections of the centroid trajectories (colored in blue) on the X-Y, X-Z, and Y-Z planes.

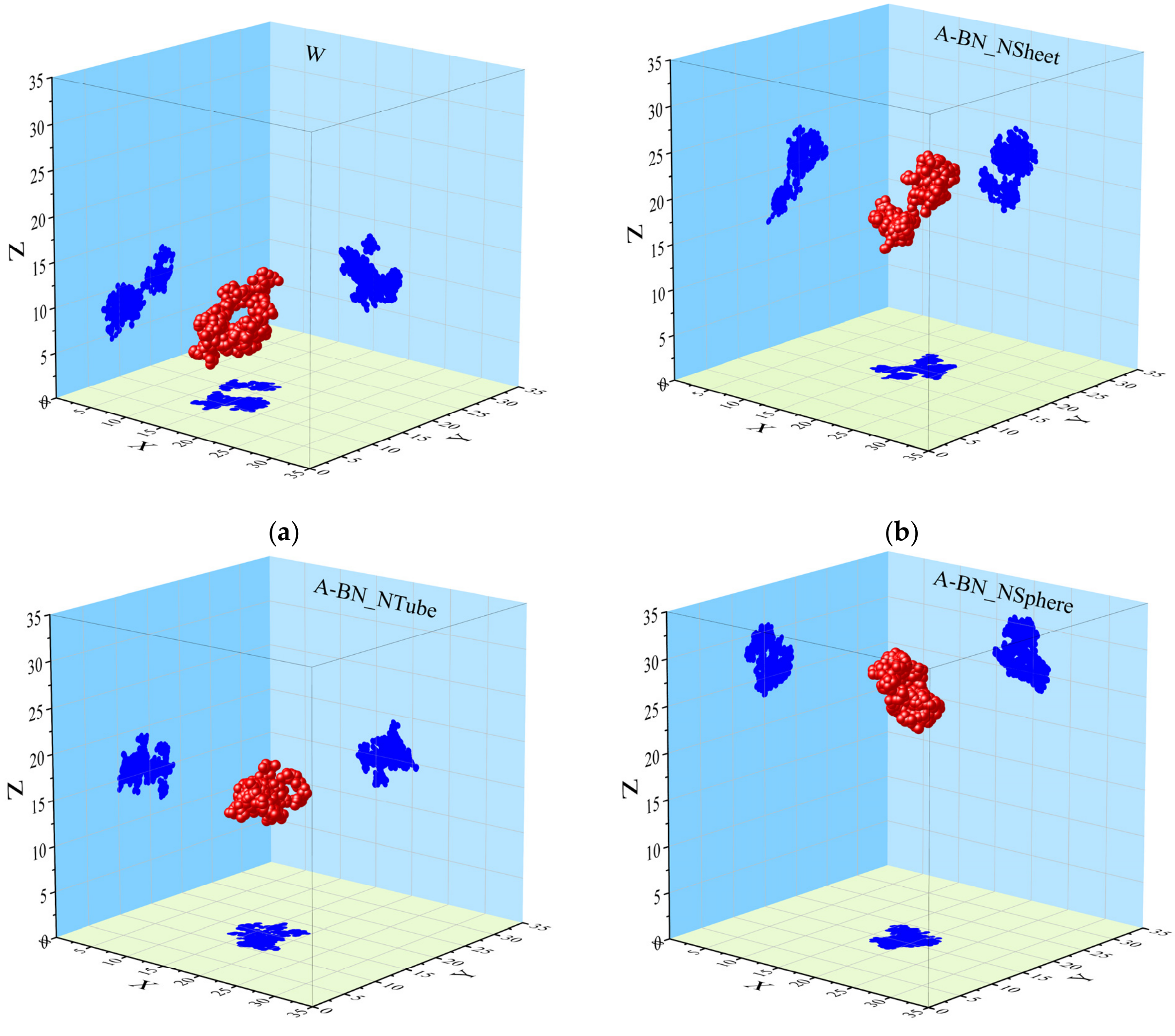

(**a**) (**b**) (**c**) (**d**)

Figure S5. Illustration of centroid trajectories of (a) W, (b) A-BN_NSheet, (c) A-BN_NTube, and (d) A-BN_NSphere models.

To examine the center of mass trajectories at a quantitative level, further calculations focused on the maximum spanned distances along the X, Y, and Z directions on the three projection planes and the corresponding averages. The results are shown in Table S3. The W model showed the largest spanned distance (11.216 Å), whereas the A-BN_NSphere model exhibited the smallest value (7.171 Å, corresponding to a decrease of 56.41%). This indicates that the MO doped with A_BN_NSphere nanoparticles can effectively restrict the movement of water molecules and reduce their diffusion range. Additionally, the maximum spanned distances in the X, Y, and Z directions in the A-BN_NSphere model were not much different, with a difference of only 1.88% between the maximum and minimum values, compared to 34.44% in the W model. This shows that the diffusion of water molecules in the A-BN_NSphere model was not only smaller in range but also more uniform, primarily involving movements around the surface of the doped A-BN_NSphere nanoparticles. This uniformity helps to effectively reduce the aggregation of water molecules into clusters, thus reducing the probability of the "small bridge" effect. Consequently, the aging of the transformer insulation system can be slowed down, ensuring its safe and stable operation.

Table S3. Maximum spanned distances of projected point in the plane of projection.

**Model**	**W**	**A-BN_NSheet**	**A-BN_NTube**	**A-BN_NSphere**
X-direction	8.343	7.563	8.056	7.171
Y-direction	11.216	9.516	8.561	7.137
Z-direction	8.441	9.416	7.368	7.306
average value(Å)	9.333	8.317	7.995	7.205

S2.3. The definitions of hydrogen bond

The definitions of hydrogen bonds are based on energetic and geometric criteria, with the latter being commonly used in molecular dynamics. The definition of hydrogen bond involves three atoms; a highly electronegative atom X can form a chemical bond with an H atom, with the formed X–H being the donor of the hydrogen bond; Y represents another highly electronegative atom, which serves as the hydrogen bond acceptor. According to the geometric criterion, a hydrogen bond is formed when the distance R_{H-Y} between the H atom and the acceptor is less than 2.5 Å, and the angle θ between X–H and Y is greater than 100°, as illustrated by the dashed line in Figure S6.

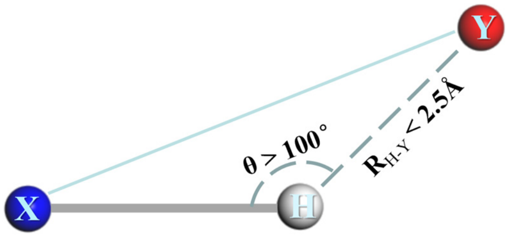

Figure S6. Geometric criterion for hydrogen bond formation.

S2.4. Schematic diagram of hard-ball probe model.

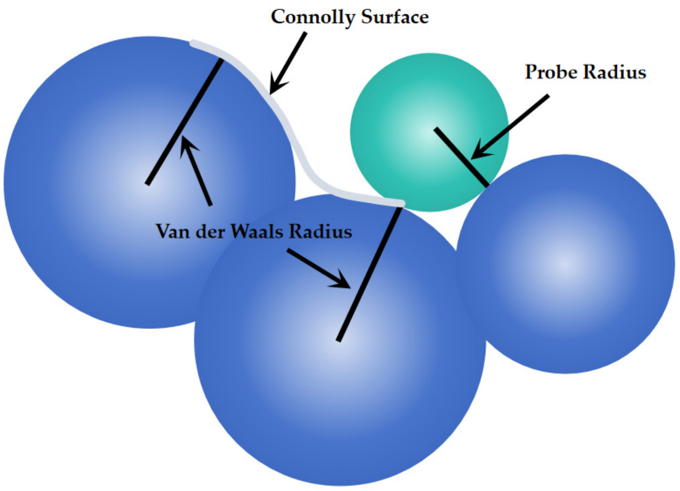

Figure S7. Schematic diagram of hard-ball probe model.

S2.5. The FFV in different types BN-doped MO models

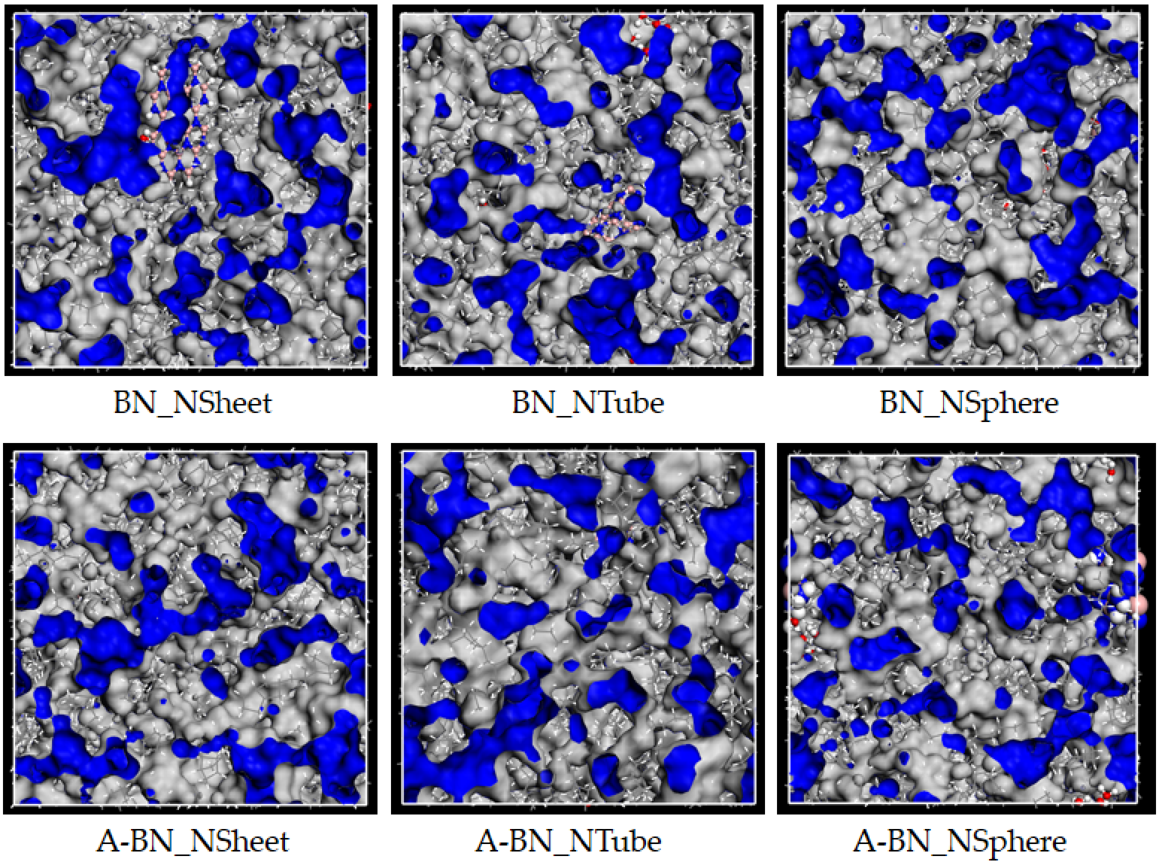

Figure S8. Visualized FFV calculation results in different types BN-doped MO models.

S2.6. The hydrogen bonding between the grafted amino groups on the surface of the BN nanospheres.

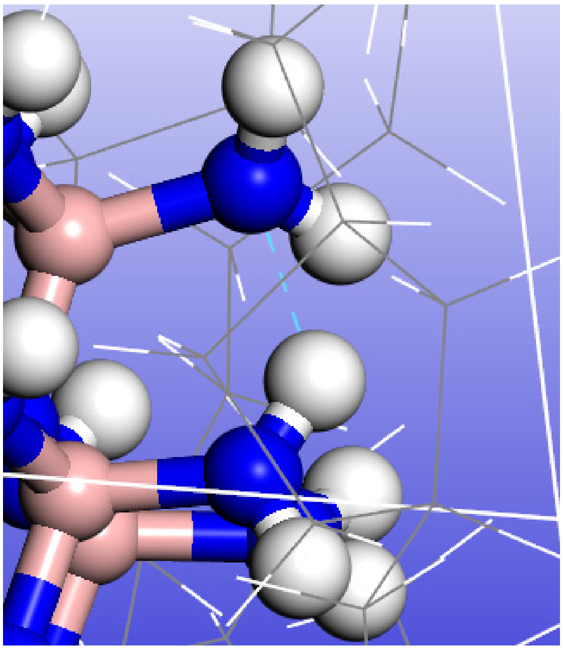

Figure S9. The hydrogen bonding between the grafted amino groups on the surface of the BN nanospheres (N-H···N hydrogen bonding)

S2.7. The FFV in different temperatures.

The free volume of the model gradually increased with the temperature, while the occupied volume decreased. The total volume remained almost constant, with only a slight increase due to the temperature. A larger free volume in the model provides more "holes" for the movement of water molecules in the oil, which provides the necessary conditions for their diffusion. This indicates that increasing the temperature leads to a faster diffusion of water molecules in the model, along with a weaker binding effect of BN nanoparticles on water molecules in oil. The calculated free volume-related data and trends are shown schematically in Figure S10 and the single-frame visualized FFV calculation results at different temperature in A-BN_NSphere model is shown in Figure S11.

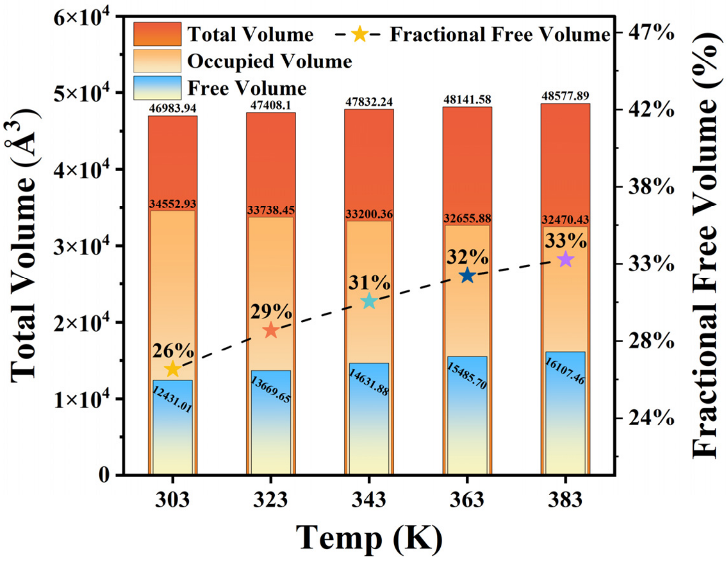

Figure S10. Correlation diagram of FFV data at different temperatures.

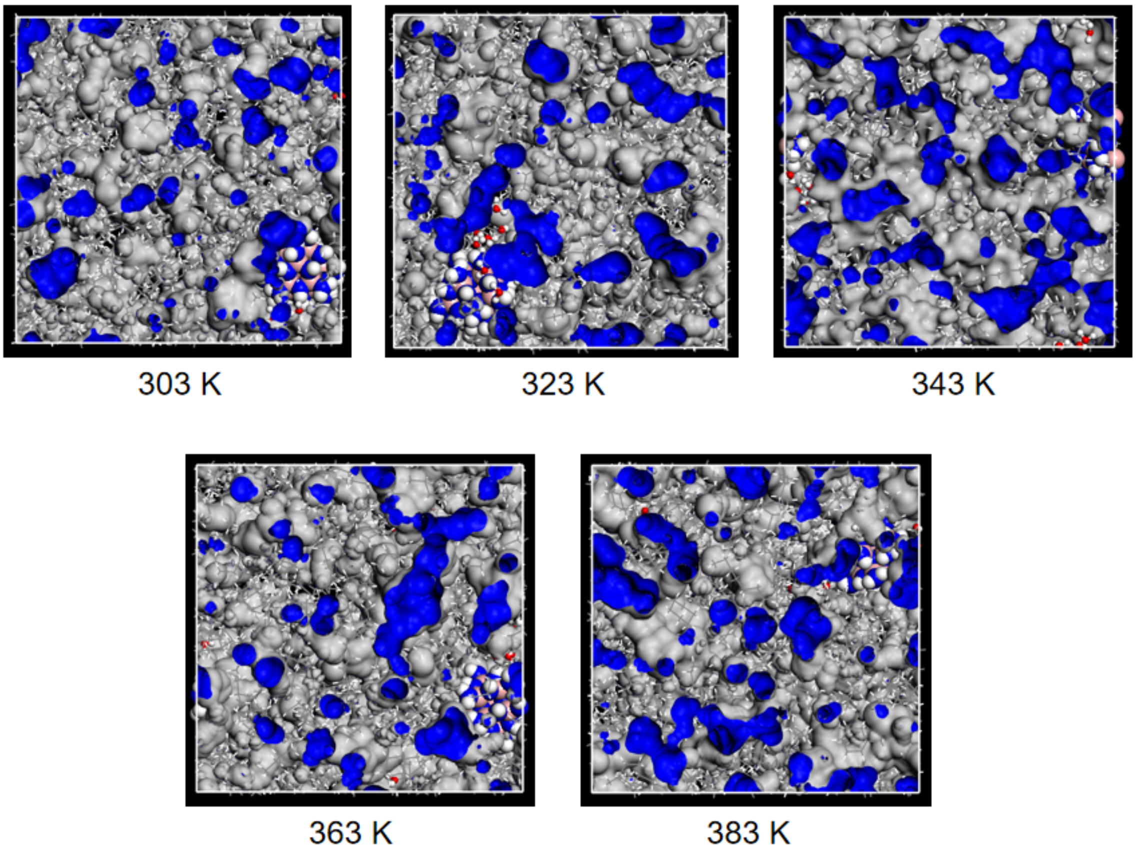

Figure S11. Visualized FFV calculation results in different temperature (in A-BN_NSphere model).

S2.8. The Calculation result of three types of hydroxylated-BN doped-MO models

It can be seen from Figure S12 and Table S4 that among the BN nanoparticles treated with hydroxylation, the doped nanospheres still show the lowest diffusion coefficient of water molecules, which is consistent with the conclusion of amination of BN in this paper. It is worth noting that compared with hydroxylation, the effect of amination on nanosheets is significantly different, and the diffusion coefficient of amination BN nanosheets is better than that of hydroxylation BN nanosheets. However, for nanotubes and nanospheres, there is little difference in their performance after hydroxylation and amination.

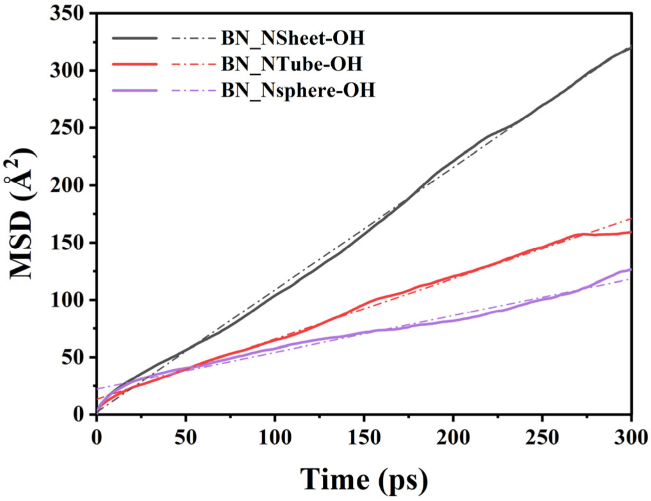

Figure S12. MSD curves of water molecules in the hydroxylation models.

Table S4. Fitting slopes of MSD curves and diffusion coefficients of water molecules in the hydroxylation models.

Model	BN_NSheet-OH	BN_NTube-OH	BN_Nsphere-OH
a	1.0690	0.5272	0.4106
D (Å^2/ps)	0.1782	0.0879	0.0684

We also calculated the interaction energy between water molecules and MO, as shown in Table S5. The results show that the interaction energy between water molecules and MO in BN_NSphere-OH model is significantly higher than that in BN_NSheet-OH model. This strong interaction results in the lowest diffusion coefficient of water molecules in the BN_NSphere-OH model.

Table S5. Interaction energy between water molecules and MO in the hydroxylation models.

Model	BN_NSheet-OH	BN_NTube-OH	BN_Nsphere-OH
E_{total} (kcal/mol)	3644.1858	3621.4817	3841.3922
E_{MO} (kcal/mol)	3701.3360	3699.5263	3928.4203
E_{water} (kcal/mol)	-38.8575	-49.2839	-54.2840
E_{inter} (kcal/mol)	-18.2927	-28.7607	-32.7441
